# Self-medication of attention-deficit/hyperactivity disorder symptoms with psychoactive substances in adults: a scoping review of how symptom-relief motives are framed and operationalised across substances

**DOI:** 10.3389/fpsyt.2026.1966206

**Published:** 2026-09-30

**Authors:** Marios Adamou, Christos Petrou

**Affiliations:** 1 School of Human and Health Sciences, University of Huddersfield, Huddersfield, United Kingdom; 2 Department of Health Sciences, School of Life and Health Sciences, University of Nicosia, Nicosia, Cyprus

**Keywords:** attention-deficit/hyperactivity disorder, cannabis, comorbidity, nicotine, non-prescribed stimulants, scoping review, self-medication hypothesis, substance use disorder

## Abstract

**Introduction:**

Adults with attention-deficit/hyperactivity disorder (ADHD) use tobacco, cannabis, alcohol, cocaine and non-prescribed stimulants far more than the general population. The self-medication hypothesis holds that people with ADHD, including those undiagnosed, use substances to relieve core symptoms rather than for recreation or shared vulnerability to addiction, and is widely invoked to reframe illicit substance use as undiagnosed ADHD. How this claim is framed and tested has not been mapped across substances.

**Objective:**

To map the literature invoking self-medication of ADHD symptoms in adults, substance by substance, and to characterise how each source frames the concept relative to misuse, recreational or shared-liability accounts.

**Eligibility criteria:**

Empirical studies, reviews, commentaries, preprints and trial-register records on self-medication of ADHD symptoms with a psychoactive substance in adults or predominantly adult samples; English; inception to 21 July 2026.

**Information sources:**

PubMed, OpenAlex and Consensus, with ClinicalTrials.gov and a preprint search. Searches were conditioned on self-medication and motive terminology, so the corpus maps the self-medication literature and does not sample the misuse or shared-liability literature independently.

**Charting methods:**

Sources were charted for substance, design, population, operationalisation of self-medication, and a framing category (self-medication-framed; mixed; misuse- or shared-liability-framed; neutral/descriptive). A descriptive map was built by substance. No critical appraisal or evidence-strength ranking was made.

**Results:**

Of 937 records identified and 820 screened, 109 sources (1986–2026) met the criteria. Coverage was heaviest for non-prescribed stimulants (n=35), cannabis (n=29), nicotine (n=25) and alcohol (n=17); no eligible ketamine source was identified. Self-medication framing was most prevalent, and uniquely accompanied by experimental data, for nicotine; prevalent but observational for cannabis; coexisted with academic and recreational motives for stimulants; and alcohol was mostly framed as comorbidity or shared liability. Self-medication was overwhelmingly inferred from association or motive report rather than tested against symptom change.

**Conclusions:**

Self-medication framing is substance-specific in prevalence and accompanying evidence. Because the search was conditioned on self-medication terminology, the map describes how the literature frames the claim and does not adjudicate between competing accounts. Applying self-medication as a blanket explanation for substance use in people with ADHD symptoms is not warranted by how the claim has been tested.

## Introduction

Attention-deficit/hyperactivity disorder (ADHD) is a common neurodevelopmental condition that persists into adulthood in a substantial proportion of cases (1, 2) Psychiatric and physical comorbidity is the rule rather than the exception: across representative samples the majority of adults with ADHD meet criteria for at least one additional psychiatric disorder (3), and pooled analyses give markedly elevated odds of mood, anxiety and substance use disorders relative to adults without ADHD (4). In a nationwide population cohort, adults with ADHD showed sharply raised prevalence of psychiatric conditions and a roughly two-fold excess of metabolic conditions (5).

Among these comorbidities, substance use disorder (SUD) is of particular relevance to this review. The association between ADHD and substance use is consistent across tobacco, alcohol, cannabis and stimulants and is observed in two directions. In prospective ADHD cohorts, childhood ADHD predicts later substance use and dependence (6, 7) and in the longitudinal MTA follow-up, weekly cannabis use and daily smoking were substantially more common in ADHD than in controls (8). In young men, ADHD raised the odds of initiating amphetamine and non-medical stimulant use (9). In the reverse direction, ADHD is over-represented among people in treatment for SUD: the International ADHD in Substance Use Disorders Prevalence study identified adult ADHD in a substantial minority of treatment-seeking SUD patients across ten countries (10, 11).

Several mechanisms could generate this association. The self-medication hypothesis (SMH), articulated by Khantzian (12, 13) proposes that people with a psychiatric disorder use substances to relieve specific symptoms, that the substance chosen is not random but reflects its pharmacology, and that treating the underlying disorder should reduce the substance use. That last prediction has itself been tested for ADHD, where stimulant treatment in youth did not increase, and may reduce, later SUD (14). Applied to ADHD, the SMH predicts that people with ADHD, including those not yet diagnosed, use stimulant-like or arousal-modulating substances (nicotine, cocaine, non-prescribed stimulants, caffeine) to improve attention and reduce restlessness. Competing accounts attribute the same association to behavioural disinhibition and impulsivity, to a shared genetic or familial liability to both ADHD and addiction, to comorbid conduct and antisocial pathology, and to ordinary social and recreational determinants of substance use.

The distinction is important well beyond nosology. In clinical and lay settings, the self-medication account is increasingly used to reframe illicit substance use as an unmet treatment need arising from undiagnosed ADHD. This framing is not baseless: among regular illicit psychostimulant users, a large fraction screen positive for adult ADHD while only a small minority have ever been diagnosed, so a genuine reservoir of undiagnosed ADHD does exist in substance-using populations (15). But the same observation is equally consistent with shared liability and with ADHD being a marker rather than the motive for use. The framing can be compassionate and clinically useful, yet it can also function as a rationalisation that forecloses assessment of recreational use, dependence, and shared liability. Adjudicating it requires evidence that goes beyond a correlation between ADHD and substance use: it requires that use be linked to symptom relief specifically, ideally prospectively and against measured symptoms.

The evidence bearing on this question is heterogeneous by design (surveys, qualitative studies, neuroimaging, trials, commentaries), scattered across substances, and conceptually inconsistent in how “self-medication” is defined and measured. This is the configuration a scoping review is designed to address: to map the extent, range and nature of the evidence, to clarify how the concept is operationalised, and to identify gaps, rather than to pool effects or grade effectiveness. No prior review has mapped the self-medication evidence across the full range of substances in adults, including emerging agents such as ketamine and classic psychedelics.

## Aim

This scoping review maps the empirical and conceptual literature on self-medication of ADHD symptoms with psychoactive substances in adults, and characterises, for each substance, how that literature frames and operationalises the self-medication concept.

The primary review question was: What is the nature and extent of the literature that invokes self-medication of ADHD symptoms by adults with ADHD (diagnosed or undiagnosed) using psychoactive substances, and how does this literature differ by substance? Subquestions addressed which substances are implicated; how self-medication is framed relative to competing accounts (misuse, recreational use, shared liability); how self-medication is operationalised and measured; and the principal gaps, including under-studied substances.

## Method

This scoping review followed updated JBI methodological guidance (16), drew on the framework proposed by Arksey and O’Malley (17), and is reported in accordance with the Preferred Reporting Items for Systematic Reviews and Meta-Analyses extension for Scoping Reviews (PRISMA-ScR) (18). An *a priori* protocol was prepared and is available as a **Supplementary File**; because PROSPERO does not register scoping reviews, the protocol was not registered on that platform. Deviations from the protocol are described below and in the audit ledger accompanying this review.

The review addressed a single question: what is the nature and extent of the literature invoking self-medication of ADHD symptoms by adults with ADHD, whether diagnosed or undiagnosed, using psychoactive substances, and how does that literature differ by substance? The question was framed widely across substance, study design and document type, because the object of the review is the shape and distribution of a heterogeneous and partly conceptual literature rather than the effect of a single intervention. Following the population–concept–context (PCC) structure recommended for scoping reviews, the population was adults, or predominantly-adult samples, with ADHD or ADHD symptoms; the concept was the self-medication of ADHD symptoms with a psychoactive substance, including how sources frame it relative to misuse, recreational use or shared liability; and the context was unrestricted by setting or country.

Three bibliographic sources were searched: PubMed (NCBI Entrez), OpenAlex and Consensus. These were supplemented by ClinicalTrials.gov, a trial register, and a preprint search via OpenAlex, all from inception to 21 July 2026. Each search combined an ADHD population block with a concept and substance block covering self-medication terminology and every pre-specified substance class. Because every bibliographic and preprint query required self-medication or motive terminology (only the four trial-register queries did not), the corpus is conditioned on that terminology: it maps the literature that invokes self-medication and does not independently sample records framed solely as misuse, diversion, addiction or shared liability. This is a design property of the review, not an incidental limitation, and it is the reason the review characterises framing rather than adjudicating between competing accounts. The Entrez interface returned truncated results on over-long combined queries, so the concept and substance block was run as a series of shorter per-substance queries; each query is logged verbatim, with its interface and hit count, in **Supplementary Table 2** and the audit ledger. PsycINFO and Embase were not searched and no backward or forward citation searching was performed; the OpenAlex and Consensus interfaces return relevance-ranked results and were capture-limited, so the corpus is not exhaustive even for the self-medication literature. Searches were restricted to English-language records, a restriction adopted for feasibility and recorded as a limitation.

Records were exported programmatically and deduplicated by normalised title and DOI, both within and across sources. Titles and abstracts were then screened against the eligibility criteria using a structured, criterion-referenced procedure applied to every record; where no abstract was available, the record was screened on its title and available metadata. A record was retained for full-text assessment where it plausibly addressed both the adult-ADHD population and the self-medication concept, or the self-medication-versus-misuse question, with an inclusive threshold at the abstract stage. Full texts were then assessed against the criteria, or the complete abstract where the full text was not accessible. Screening and assessment were carried out by a single reviewer using a large-language-model-assisted first pass with reviewer adjudication; the absence of a second independent human reviewer is a limitation, discussed below. Reports and sources were distinguished throughout, so that multiple reports of one project were linked to a single source entry. The selection process and counts are shown in the PRISMA-ScR flow diagram (**Figure 1**) (18).

**Figure 1:**
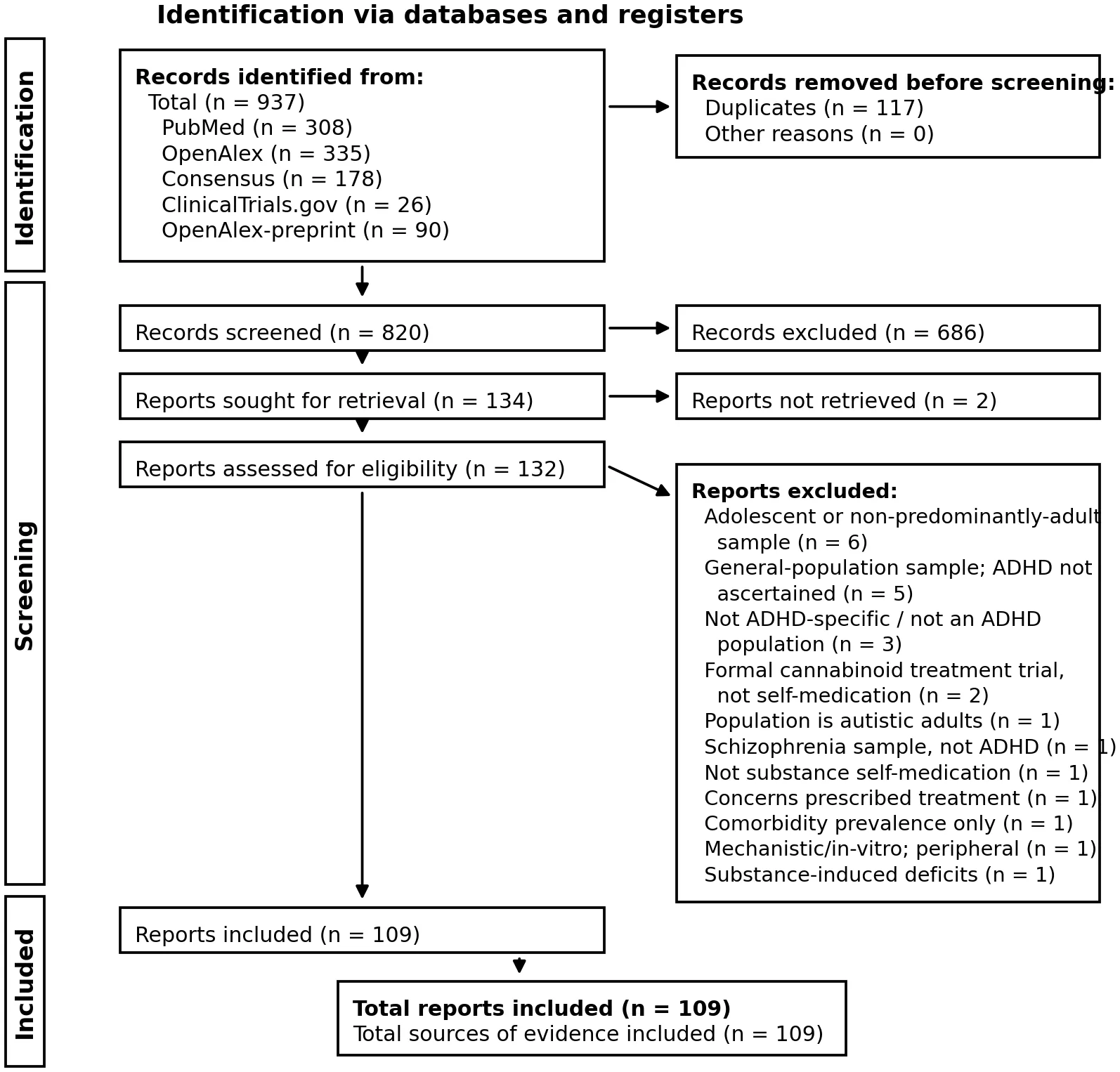
PRISMA-ScR flow diagram.

Data were charted into a structured JSON ledger, piloted on an initial set of sources and refined as charting proceeded. The charted fields were the source label and type; linked reports with identifiers and retrieval record; country or region; design or document type; population, sample size and setting; how the self-medication concept was operationalised in the source; verbatim findings with locators; and an analytic stance. Charting was performed by a single reviewer, and every included report was resolved to a DOI or PMID. No formal critical appraisal was undertaken, because the objective was to map the extent, nature and characteristics of the evidence rather than to determine effectiveness, consistent with JBI scoping-review guidance (16). The findings describe the available evidence; they do not establish the trustworthiness or comparative effectiveness of the included sources.

Analysis was a descriptive numerical summary complemented by a descriptive categorisation of framing. Each source was assigned to one of four framing categories (self-medication-framed, mixed, misuse- or shared-liability-framed, and neutral/descriptive) recording how the source characterised the ADHD–substance relationship and was tagged with the substance class or classes it addressed. These categories record framing, not evidence of benefit: a source is self-medication-framed if it presents use as symptom-directed, whether by stated motive, pharmacological rationale or symptom measurement, and the category does not distinguish these evidentiary levels, which is why no evidence-strength verdict is derived from it. Sources addressing more than one substance contribute to more than one row of the map. The framing category was assigned at the level of the source rather than the substance, and the reader should bear this in mind when interpreting the per-substance columns: a source counted under a substance it addresses only secondarily carries its source-level framing into that row. The categories were applied by a single reviewer and represent an interpretive mapping, not a validated instrument. Results are presented as a characteristics table (**Supplementary Table 1**), a substance-by-framing map (**Figure 2**; **Table 1**), and a narrative synthesis organised by substance in order of literature volume. The completed PRISMA-ScR checklist (18) is provided in **Supplementary Table 3**.

**Figure 2:**
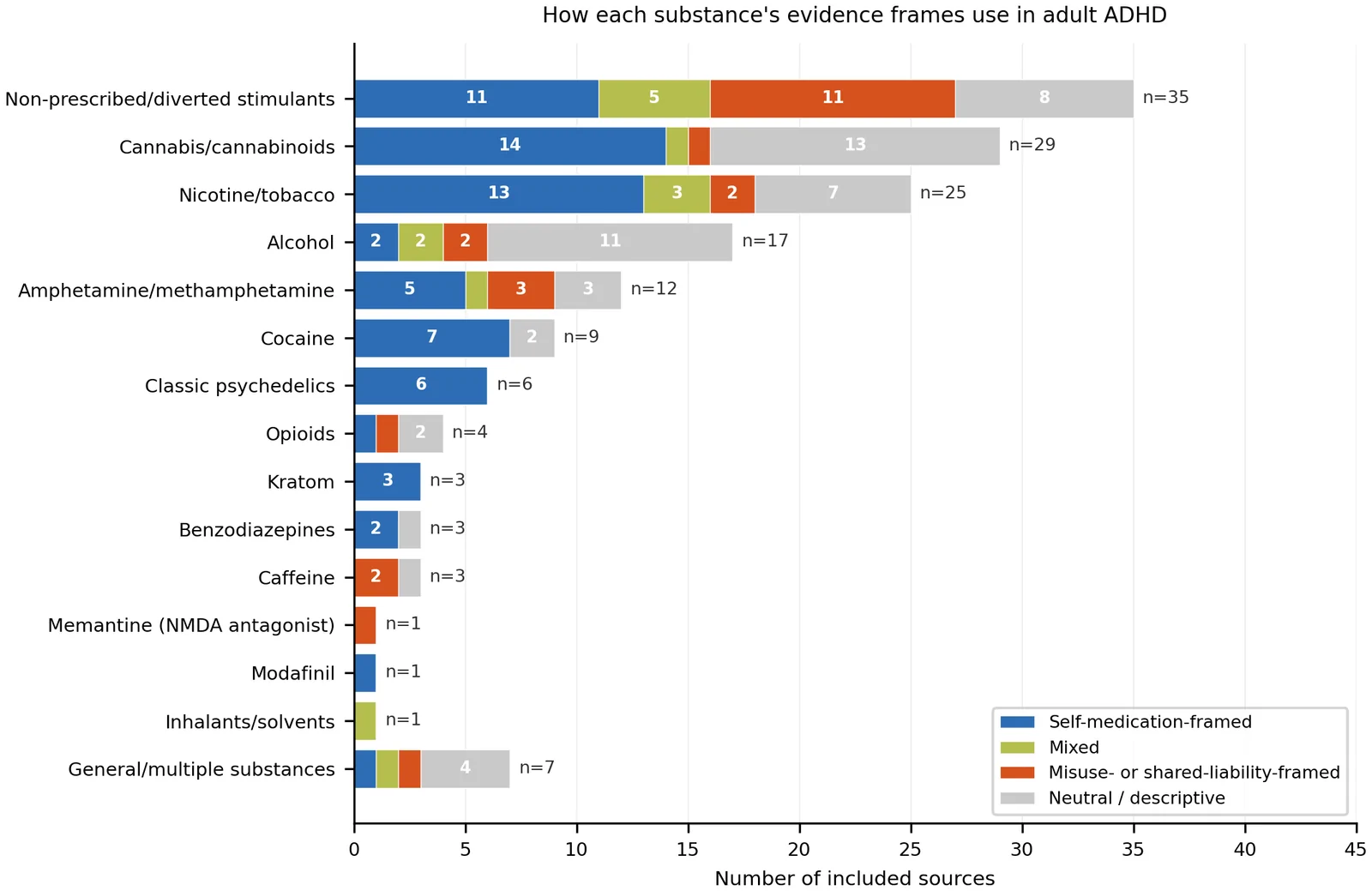
Substance-by-framing map. Each source is counted once per substance it addresses; the framing category is assigned at source level. Counts describe how sources frame the relationship and are not a measure of evidentiary strength.

**Table 1:** Distribution of framing category by substance.

Substance	Sources (n)	Self-medication-framed	Mixed	Misuse- or shared-liability-framed	Neutral/descriptive
Non-prescribed/diverted stimulants	35	11	5	11	8
Cannabis/cannabinoids	29	14	1	1	13
Nicotine/tobacco	25	13	3	2	7
Alcohol	17	2	2	2	11
Amphetamine/methamphetamine	12	5	1	3	3
Cocaine	9	7	0	0	2
Classic psychedelics	6	6	0	0	0
Opioids	4	1	0	1	2
Kratom	3	3	0	0	0
Benzodiazepines	3	2	0	0	1
Caffeine	3	0	0	2	1
Memantine (NMDA antagonist)	1	0	0	1	0
Modafinil	1	1	0	0	0
Inhalants/solvents	1	0	1	0	0
General/multiple substances	7	1	1	1	4

## Results

### Selection of sources

The searches identified 937 records across the three bibliographic sources and two supplementary sources (PubMed, 308; OpenAlex, 335; Consensus, 178; ClinicalTrials.gov, 26; OpenAlex preprint search, 90). After removal of 117 duplicates, 820 records were screened by title and abstract. Of these, 686 were excluded and 134 reports were sought for retrieval. Two reports were grey-literature/conference records for which no resolvable identifier or stable publisher locator could be found and which could not be retrieved for citable inclusion; 132 reports were assessed for eligibility. Twenty-three reports were excluded at full-text assessment. The reasons were: reporting an adolescent or non-predominantly-adult sample (n=6); sampling a general population in which ADHD was not ascertained (n=5); evaluating formal cannabinoid treatment efficacy rather than self-medication behaviour (n=2); not concerning ADHD or not being ADHD-specific (n=3, including one study in which ADHD appeared only as a vulnerability-domain score); studying self-medication in autistic adults rather than adults with ADHD (n=1); concerning a schizophrenia rather than ADHD population (n=1); not concerning substance self-medication (n=1); concerning prescribed treatment rather than self-medication (n=1); reporting comorbidity prevalence only (n=1); being mechanistic/*in-vitro* with only peripheral relevance (n=1); describing substance-induced deficits rather than ADHD self-medicated with a substance (n=1); and being a study protocol with no data (n=1). Twelve of these exclusions were made on re-screening of the initially included corpus against the population and concept gates during revision. In total, 109 sources were included (**Figure 1**). Full-text exclusions with reasons are listed in **Supplementary Table 4**.

### Overview of included sources

The 109 included sources spanned 1986 to 2026, with most published in the two most recent periods (2010–2019, n=41; 2020–2026, n=45). The dominant designs were cross-sectional surveys (n=41) and reviews (n=30, including 5 systematic reviews or meta-analyses), followed by qualitative studies (n=15, including one content analysis), experimental or trial designs (n=7), longitudinal designs (n=7, comprising 5 cohort studies and 2 intensive-longitudinal EMA or daily-diary studies), neurobiological/imaging studies (n=5), case reports (n=2), and single observational-comparative and commentary sources (n=2). Where geography was identifiable, samples were predominantly from the United States, with contributions from Brazil, Norway, Finland, Iraq, Japan, Switzerland, Latvia and Israel; many sources did not report a single country. Characteristics are summarised in **Supplementary Table 1**. The complete set of included sources, with linked references, is provided in the audit ledger and the evidence-map table. Additional charted sources spanning the substance range are indexed there (19, 20).

Across the whole corpus, 48 sources were self-medication-framed, 19 were misuse- or shared-liability-framed, 11 were mixed, and 31 were neutral or descriptive (that is, reporting an ADHD–substance association or invoking self-medication in passing without adjudicating it). The distribution of these framings differed substantially by substance (**Figure 2**, **Table 1**). Because the search was conditioned on self-medication terminology, the predominance of self-medication framing across the corpus reflects the retrieval strategy and should not be read as the balance of evidence between competing accounts.

### Substance-by-substance map

#### Non-prescribed and diverted stimulants (n=35)

This was the largest literature (21–55). Where the framing favoured self-medication, sources reported that non-medical users of prescription stimulants screened positive for ADHD symptoms at high rates and framed their use as self-treatment of attention problems (21–23, 27, 31–34, 36, 37, 55). A larger set characterised non-medical stimulant use as driven by academic performance-enhancement and recreational motives, and documented diversion and misuse patterns in college and fraternity populations in which ADHD symptomatology was not the dominant driver (24, 35, 39–42, 45, 49, 51, 53, 54). Several sources remained explicitly neutral between “misuse or self-treatment” (44, 47, 48, 50, 52). Symptom-relief and non-therapeutic motives therefore coexist in this literature, and most sources that addressed motive treated the non-therapeutic motives as at least as prominent.

#### Cannabis and cannabinoids (n=29)

Cannabis had the largest volume of self-medication-framed sources (56, 57), including qualitative and survey work in which users described therapeutic motives (symptom relief, focus, sleep) and medicinal framing of non-prescribed use, alongside emerging neuropharmacological and neuroimaging work on cannabinoid pathways (21, 27, 56–67). A substantial share of the cannabis corpus was neutral/descriptive (28–30, 68–77), reporting elevated cannabis use or problematic-use associations in ADHD without addressing a symptom-relief mechanism, and one source emphasised a misuse or shared-liability framing (78). The self-medication framing for cannabis rests largely on cross-sectional and self-report designs.

#### Nicotine and tobacco (n=25)

Nicotine was the substance for which self-medication framing was most often accompanied by experimental data (79–83). Controlled studies reported that transdermal nicotine reduced ADHD symptoms and negative mood in adults with ADHD independent of smoking status, that nicotine improved sustained attention on neuropsychological testing, and that smoking-motivation profiles in adults with ADHD loaded on cognitive-enhancement and negative-reinforcement factors (22, 23, 84–94). The proposed mechanism, nicotine acting as an indirect dopamine agonist qualitatively similar to stimulant medication, recurred across sources. A minority framed the ADHD–smoking link primarily through behavioural disinhibition, externalising comorbidity or shared liability rather than symptom relief, or argued for reverse causation from smoking to attention problems (95, 96), and several sources were descriptive associations only (25, 26, 68, 69, 97–99). Nicotine is the only substance in the corpus for which the self-medication proposition has been tested against measured symptom change, though the experimental sources are small-sample or laboratory-based.

#### Alcohol (n=17)

Alcohol was rarely framed as symptom-relief self-medication (27, 52, 53, 68–71, 74, 99–107). Most sources treated the ADHD–alcohol association as comorbidity, examined drinking motives (frequently social or coping motives rather than ADHD-symptom relief), or invoked shared-liability and internalising pathways (68–71, 74, 99, 101–103, 105, 107). Only a small minority were self-medication-framed (27, 100).

#### Amphetamine and methamphetamine (n=12)

The literature overlapped heavily with the non-prescribed-stimulant literature (23, 32–34, 108). Self-medication-framed sources, with a mechanistic rationale parallel to prescribed stimulant therapy, were the largest component, but misuse- or shared-liability-framed and neutral sources were nearly as numerous, and one study that measured motives directly found no difference in coping motives between users with and without ADHD; the corpus carried the same motive-ambiguity as the broader stimulant literature.

#### Cocaine (n=9)

The cocaine literature was small, older, and predominantly self-medication-framed (31, 32, 100, 109–112): case series, treatment-seeking-sample studies and neurobiological (dopamine-transporter) work repeatedly described cocaine use as self-medication of a presumed dopaminergic deficit in adults with ADHD or ADD-residual type. This consistency of framing should be read against the age and design of the sources (several are 1980s–1990s case reports and clinical descriptions) and the difficulty of separating self-medication from cocaine’s own reinforcing pharmacology.

#### General/multiple-substance sources (n=7)

Sources addressing substance use in aggregate (113–119) most often invoked self-medication as one candidate mechanism among several (disinhibition, shared liability, comorbidity), and on balance did not present it as the primary explanation.

#### Classic psychedelics (n=6)

All identified sources were self-medication-framed (33, 120–124): microdosing surveys, a systematic review of novel psychoactive substances, and conference reports proposing psilocybin/LSD microdosing for ADHD symptoms. All are preliminary (self-report, uncontrolled, conference-stage) and record user-reported symptom-management intent rather than demonstrated efficacy.

#### Opioids (n=4)

A small literature (30, 35, 101, 108) in which ADHD appeared as a prevalent comorbidity in opioid-treatment populations; the framings were divided (one source self-medication-framed, one misuse- or shared-liability-framed, two neutral), on limited and indirect evidence.

#### Kratom (n=3)

All three sources were self-medication-framed (62, 125, 126), describing use for focus, energy, and self-treatment of psychiatric symptoms including ADHD, within broader self-medication survey work. One is a case report of cardiotoxicity in a patient using kratom at high daily doses; its self-medication framing reflects the patient’s stated motive while the reported outcome was harm, which illustrates directly that the framing category records motive rather than benefit.

#### Caffeine (n=3)

Sparse (40, 127, 128), and no source was self-medication-framed. The one study that directly tested whether caffeine consumption tracks ADHD symptoms found no association and concluded that caffeine use does not represent self-medication; the remaining sources treat caffeine as a cause of psychiatric symptoms or describe its use without adjudicating motive.

#### Benzodiazepines (n=3), modafinil (n=1), inhalants/solvents (n=1) and memantine (n=1)

Each was represented by very few sources. Benzodiazepine and modafinil sources were self-medication-framed; the single Japanese solvent source described organic solvents chosen to cope with secondary ADHD-related symptoms rather than to directly improve core symptoms. The single memantine source (a distinct NMDA-receptor antagonist with an Alzheimer’s-disease indication, not a controlled dissociative) (129) analysed online self-reports of off-label use including for ADHD and was misuse- or shared-liability-framed.

#### Ketamine (n=0)

Ketamine was a pre-specified substance class because it is increasingly discussed as a candidate in this context, but no eligible source was identified: the only record retrieved was a hypothesis paper on neuroticism and internalising disorders that does not concern ADHD and was excluded at full-text assessment. The absence of any eligible ketamine source is itself a finding: the assumption that ketamine use reflects ADHD self-medication is, at present, unstudied in the literature this search retrieved.

A cross-cutting observation is methodological: in the large majority of included sources, “self-medication” was inferred from a cross-sectional association between ADHD symptoms and substance use, or from users’ retrospective motive reports, rather than tested by linking use to prospective change in measured ADHD symptoms. The small number of experimental sources (chiefly the nicotine-patch studies) are the principal exception.

## Discussion

### Summary of main findings

This review mapped 109 sources published over four decades that invoke self-medication of ADHD symptoms in adults. The literature is substantial in volume but concentrated on four substances (non-prescribed stimulants, cannabis, nicotine, and alcohol), dominated by cross-sectional and qualitative designs, and heavily weighted towards recent years. The central finding is that self-medication framing is substance-specific in both its prevalence and the kind of evidence that accompanies it. For nicotine, the framing is accompanied by controlled data on symptom and mood change. For cannabis, and to a lesser extent cocaine and amphetamine-type stimulants, the framing is common but rests on observational and self-report designs. For non-prescribed stimulants, self-medication framing coexists with academic and recreational motives that most sources treat as at least as prominent. For alcohol, sources predominantly frame the association as comorbidity or shared liability. Opioids, kratom, classic psychedelics, benzodiazepines, caffeine and modafinil are each represented by too few sources to characterise beyond the framing recorded in **Table 1**, and no eligible ketamine source was identified.

### Self-medication framing differs across substances

The studies suggest that the pattern of self-medication tracks pharmacology and study design more than it tracks a single unifying mechanism. Substances with a direct, plausible action on the dopaminergic and attentional systems implicated in ADHD (nicotine, stimulants, cocaine) attract self-medication framing and, for nicotine, experimental testing. Substances whose association with ADHD is more often attributed to shared externalising liability or comorbidity, alcohol most clearly, attract less symptom-relief framing even when the raw association is strong. A positive ADHD–substance association is necessary but not sufficient for a source to frame use as self-medication. The substances for which the claim is made most confidently in clinical and lay discourse, the illicit stimulants, are the ones where the sources themselves are most divided about motive.

### The gap between assertion and test

Across the corpus, self-medication was far more often asserted or inferred by the use than tested. The main design, a cross-sectional correlation between ADHD symptoms and substance use sometimes accompanied by self-reported motives, cannot distinguish symptom-relief use from disinhibition, shared liability, or reverse causation, in which substance use produces attentional symptoms (6). Where the concept was operationalised rigorously (measured symptoms, prospective design, placebo control, or motive instruments validated against symptom change), the evidence base thins considerably. For most substances the hypothesis remains plausible but under-tested. That conclusion is consistent with the long-standing critique that the self-medication hypothesis has been influential in clinical culture out of proportion to its empirical support (130). It follows that using “self-medication of undiagnosed ADHD” as a blanket account for illicit substance use is not warranted by the current evidence; the claim can be reasonable for specific substances and specific patients but should be treated as a hypothesis to be assessed, not a default explanation.

### Implications for clinical reasoning

Because this review maps framing without critical appraisal and its search was conditioned on self-medication terminology, it offers no basis for treatment or prescribing decisions, which lie outside its question and its search. One observation does follow from the map. Across the corpus, self-medication was inferred from association or retrospective motive report far more often than it was tested, so a patient’s report of “self-medicating” ADHD symptoms is a reason to assess for ADHD, not evidence that ADHD is present or that the substance use is symptom-directed. A substantial reservoir of undiagnosed ADHD exists in substance-using populations (15) and ADHD is over-represented among people in treatment for substance use disorder (10, 11). The case for structured diagnostic assessment against DSM or ICD criteria, developmental history and corroboration is therefore strong, and it does not depend on the self-medication hypothesis being true. Whether ADHD pharmacotherapy alters substance use in adults was not addressed by this review and requires a separate systematic review of treatment outcomes.

### Research implications

The map identifies where the underlying question is live and testable. The most fundamental gap is methodological: searches not conditioned on self-medication terminology, so that the balance between self-medication and competing accounts can be assessed rather than assumed. Beyond that, priority gaps include prospective, symptom-linked designs that test whether substance use tracks ADHD symptom fluctuation rather than merely co-occurring with an ADHD diagnosis; motive measurement that distinguishes symptom relief from performance-enhancement and recreation; and treatment studies that test directly whether initiating ADHD pharmacotherapy changes substance use in adults. Basic descriptive work is also needed on substances that are widely discussed but essentially unstudied in this frame, especially ketamine and the classic psychedelics, where current sources are uncontrolled self-reports or absent.

### Strengths and limitations

The review’s strengths are a broad, pre-specified substance scope (fifteen substance classes, of which fourteen were represented by at least one eligible source), a multi-source search logged verbatim, a transparent audit ledger linking every included source to its framing category, and an explicit substance-by-framing map. Its limitations are specific and consequential, and the first is a design property rather than an incidental shortcoming. Every bibliographic and preprint query required self-medication or motive terminology, so the corpus is conditioned on that terminology: it maps the literature that invokes self-medication and does not independently sample records framed solely as misuse, diversion, addiction or shared liability. The review therefore cannot, and does not, adjudicate whether self-medication or a competing account better explains the ADHD–substance association; the predominance of self-medication framing in the corpus is in part an artefact of retrieval. PsycINFO and Embase were not searched, no backward or forward citation searching was performed, and the OpenAlex and Consensus interfaces returned relevance-ranked results that were capture-limited, so the corpus is not exhaustive even for the self-medication literature. A single reviewer, assisted by a large language model, performed the screening, charting and framing categorisation; there was no second independent human reviewer, so selection and classification error are not quantified. The framing categories are an interpretive device, not a validated instrument, and the reviewer applied them largely from titles and abstracts rather than full texts. The self-medication-framed category records that a source presents use as symptom-directed and does not distinguish stated motive from measured symptom change or from demonstrated benefit; a case report of harm can be self-medication-framed. Substance tags were derived programmatically and a source addressing several substances contributes to several rows of the map, so the per-substance columns count sources that mention a substance, not studies dedicated to it; the framing is assigned at source level, which can misrepresent a substance discussed only secondarily. During analysis this produced identifiable errors that were corrected: a memantine self-report study was initially grouped with ketamine, and three sources that failed the population or concept criteria (a schizophrenia nicotine review, a non-ADHD ketamine hypothesis paper and a meta-analysis of formal cannabinoid treatment) were identified on re-examination and excluded. A subsequent re-screening of every included source against the population and concept gates, using retrieved abstracts, publication metadata and full text where available, identified twelve further sources that did not meet them: five adolescent or non-predominantly-adult samples, five general-population samples in which ADHD was not ascertained, one study in which ADHD appeared only as a vulnerability-domain score, and one formal cannabinoid treatment trial. These were excluded and the counts, table and figures recomputed. The same re-screening corrected the framing of three sources whose own conclusions did not support self-medication and removed substance tags from three sources in which a substance appeared only as a covariate or route of administration. This re-screening was performed by the same reviewer with large-language-model assistance; it does not substitute for independent duplicate screening by a second human reviewer, and residual misattributions cannot be excluded. The search was limited to English-language records; two grey-literature records could not be retrieved with a citable identifier and were excluded. Finally, because this is a scoping review, no critical appraisal was undertaken, and no evidence-strength ranking is made: the map describes the extent and nature of the literature and must not be read as establishing effectiveness or the truth of the self-medication hypothesis for any substance.

## Conclusion

The literature invoking self-medication of ADHD symptoms by adults is substantial but uneven, and no single claim covers all of it (131). For nicotine alone the self-medication proposition has been tested against measured symptom change in controlled studies, though findings regarding motivation remain mixed. For cannabis the framing is common but observational (132), and cocaine and amphetamine/methamphetamine are framed similarly on smaller and older literatures (133). For non-prescribed stimulants and alcohol, most sources treat non-therapeutic motives, comorbidity or shared liability as at least as prominent as symptom relief (134). Opioids, kratom, classic psychedelics, benzodiazepines, caffeine and modafinil each rest on a handful of sources, too few to characterise beyond their recorded framing, and no eligible ketamine source was identified. What holds across almost the whole literature is that self-medication has been inferred from association or motive report rather than tested against symptom change.

The map describes how the literature frames the self-medication claim; because the search was conditioned on self-medication terminology, it does not adjudicate between self-medication and competing accounts, a question that requires an unconditioned search. What it does show is that the claim is far more often asserted than tested, so reframing substance use as self-medication of undiagnosed ADHD is defensible only as a substance- and patient-specific hypothesis to be assessed, not as a blanket explanation. Future research should prioritise searches that sample the misuse and shared-liability literature independently, prospective symptom-linked designs, motive measurement that distinguishes symptom relief from performance-enhancement and recreation, and basic descriptive work on substances such as ketamine and the classic psychedelics that are increasingly discussed in this context but scarcely studied.

## Protocol

An *a priori* protocol was finalised before the searches were executed on 21 July 2026 and accompanies this review as **Supplementary Data Sheet 1**. It was not registered on PROSPERO, which does not accept scoping reviews, and no alternative registry was used. Two amendments were made after the search and are recorded in the protocol: on revision, the review question was narrowed from adjudicating between self-medication and competing accounts to characterising how self-medication is framed within the literature that invokes it, because the search was conditioned on self-medication terminology; and every included source was re-screened against the population and concept criteria, leading to twelve further exclusions and six recodings, as reported in the Results and Limitations.

## Reviewer contributions and process

Source identification, screening, charting and synthesis were conducted by a single reviewer with large-language-model assistance, as described in the Method and Limitations. This is a methodological limitation relative to the dual-independent-reviewer standard.

## Data Availability

The original contributions presented in the study are included in the article/**Supplementary Material**. Further inquiries can be directed to the corresponding author/s.

## Supplementary material

Supplementary Table 1 Characteristics of all 109 included sources: authors and year, identifier, design, substance(s), framing category, sample size, country, setting and population, age structure, ADHD ascertainment, how self-medication was operationalised, and whether full text or abstract only was available (supplied as a spreadsheet).

Supplementary Table 2 Full electronic search strategies, with each executed query, its interface and its hit count.

Supplementary Table 3 Completed PRISMA-ScR checklist.

Supplementary Table 4 Full-text exclusions with reasons. The audit ledger (searches, flow counts, included-source charting and full-text exclusions), the screening decisions, the evidence-map data and the analysis code are provided as additional **Supplementary Files**.
