# Supplementary material for: Self-medication of attention-deficit/hyperactivity disorder symptoms with psychoactive substances in adults: a scoping review of how symptom-relief motives are framed and operationalised across substances
Source: Front Psychiatry. 2026 Sep 30;17:1966206. doi: 10.3389/fpsyt.2026.1966206 (PMC13662167; doi:10.3389/fpsyt.2026.1966206)
Supplement: Supplementary file 1 [file Table1.docx]

# Supplementary Data Sheet 1. A priori review protocol

Review title: Self-medication of ADHD symptoms with psychoactive substances in adults: a scoping review of how symptom-relief motives are framed and operationalised across substances (original working title: Self-medication versus misuse in adults with ADHD: a scoping review)

Protocol status: finalised prospectively before searching and screening (version 1.0); not registered, because PROSPERO does not accept scoping-review registrations and no alternative registry was used. Amendments after the search are recorded in the final section of this document (version 1.1, revision of September 2026). Coverage: inception to 21 July 2026. Searches were executed on 21 July 2026.

## 1. Review question

What is the nature, extent, and range of evidence describing self-medication of ADHD symptoms with psychoactive substances in adults, and to what extent does that evidence support a genuine self-medication interpretation as distinct from substance misuse or a shared-liability relationship, broken down by substance?

### Subquestions

- Which substances have been studied in relation to self-medication of ADHD symptoms in adults, and how much evidence exists for each?
- What evidence supports a self-medication interpretation (symptom-relief motives, neurocognitive/pharmacological plausibility, symptom-contingent use, improvement on use)?
- What evidence undercuts or complicates it and instead supports misuse, diversion, hedonic use, or shared genetic/environmental liability?
- How is self-medication operationalised and measured across sources?
- Where are the evidence gaps (substances, populations, designs) with explicit attention to ketamine and classic psychedelics?

## 2. Population, concept, context (PCC)

Population: Adults (>=18y) or predominantly-adult samples with ADHD/ADD by clinical diagnosis, structured interview, registry, or validated self-report (ASRS, WURS).

Concept: Self-medication of ADHD symptoms with psychoactive substances, and the analytic distinction between genuine self-medication and misuse/SUD/shared liability ('abuse masquerading as self-medication').

Context: Any setting (community, clinical, addiction/psychiatric services, student, forensic, online, harm-reduction); any country.

## 3. Eligibility criteria

### Inclusion

- Adults >=18 or predominantly-adult samples (>=80% adult or mean age >=18) with ADHD ascertained by diagnosis, interview, registry, or validated self-report
- Explicitly addresses self-medication / symptom-relief motive for ADHD with >=1 psychoactive substance, OR explicitly tests self-medication vs misuse/shared liability
- Empirical studies (quant/qual/mixed), reviews, guidelines, topical commentaries/editorials, theses, conference abstracts with resolvable identifiers
- Any context/country; inception to 2026-07-21; English

### Exclusion

- Exclusively paediatric (<18) with no separable adult data
- Animal-only studies
- No ADHD ascertainment in the sample
- Substance use in ADHD with no reference to self-medication or symptom-relief motive
- ADHD pharmacotherapy adherence or prescription-stimulant treatment-efficacy studies
- Non-English with no English full text

Source types eligible: study, review, guideline, opinion/commentary, preprint, trial register record, thesis, conference abstract.

Publication status eligible: published, in-press, preprint, trial register, grey literature.

Date span: inception to 2026-07-21. Languages: English.

### Rationales for the criteria

- adults only: Review concerns adult self-medication; adult data must be separable or predominant.
- concept gate: Keeps the map on the self-medication construct, not general ADHD-SUD comorbidity.
- english only: Single-reviewer feasibility; declared as a language-bias limitation.
- no design filter: Scoping reviews map all evidence types; recall protected.
- grey literature: Reduces publication-bias distortion of the map.

## 4. Information sources and search strategy

Planned sources: PubMed (NCBI Entrez E-utilities), OpenAlex, Consensus, ClinicalTrials.gov, and a preprint search via OpenAlex. Each search combined a fixed ADHD population block with a concept/substance block of the form (ADHD population) AND (substance term(s)) AND (self-medication/motive term(s)). Every executed query, its interface and its hit count are reported verbatim in Supplementary Table 2 and in the audit ledger (Supplementary Data Sheet 2). Records were exported programmatically and de-duplicated by normalised title and DOI, within and across sources.

## 5. Selection process

Title and abstract screening: Single AI reviewer (Claude Science). LLM-assisted structured classification (include/exclude/uncertain + reason) per abstract; reviewer adjudicates all 'uncertain' and audits a sample of excludes. No independent duplicate screening.

Full-text assessment: Single AI reviewer; full texts/abstracts via PMC and open-access DOI resolution.

Conflict resolution: Not applicable (single reviewer); no third-reviewer adjudication claimed.

Reports and sources were distinguished throughout, so that multiple reports of one project were linked to a single source entry.

## 6. Data charting

Charting: Single AI reviewer; form piloted on 2-3 diverse sources.

Planned charted fields: source label and type; linked reports with identifiers and retrieval record; country or region; design or document type; population, sample size and setting; how the self-medication concept was operationalised; verbatim findings with locators; and an analytic stance.

## 7. Analysis and presentation

Descriptive numerical summary complemented by a light descriptive categorisation. Each source was to be assigned to one of four analytic-stance categories and tagged with the substance class or classes it addressed; sources addressing more than one substance contribute to more than one row of the evidence map, and the stance is assigned at source rather than substance level.

### Pre-specified analytic-stance categories

- supports self-medication
- supports misuse-or-shared-liability
- mixed
- neutral-descriptive

Planned outputs: a characteristics table, a substance-by-stance evidence map presented as a figure and a table, and a narrative synthesis organised by substance.

## 8. Critical appraisal

Not conducted. Objective is to map evidence nature/extent and how self-medication is used and measured, not to evaluate effectiveness (JBI guidance).

## 9. Consultation exercise

Consultation: Not conducted. The optional consultation stage of the Arksey and O'Malley framework was not undertaken.

## 10. Reporting

Reporting in accordance with PRISMA-ScR; the completed checklist is provided as Supplementary Table 3.

## 11. Deviations from the protocol

Two deviations arose during execution and are reported in the manuscript. First, the NCBI Entrez interface returned no result for the full single combined query, so the concept and substance block was executed as one direct self-medication search plus one search per substance family (14 PubMed queries in total). Second, the relevance-ranked interfaces (OpenAlex, Consensus, OpenAlex preprint) were capture-limited per query, so the counts entering the flow diagram for those sources are the records captured and exported rather than the interface totals. No changes were made to the review question, the eligibility criteria or the analytic-stance categories after screening began.

**12. Amendments after the search (version 1.1, September 2026)**

Two amendments were made during peer-review revision, after searching, screening and charting had been completed on the version 1.0 criteria. Neither altered the eligibility criteria, the search or the charting fields; both are reported in the manuscript.

Amendment 1 (review question). The question was narrowed from adjudicating, for each substance, whether the evidence supports a self-medication account or is better characterised as misuse, recreational use or shared liability, to characterising how the literature that invokes self-medication frames and operationalises the concept. The reason is that every bibliographic and preprint query required self-medication or motive terminology, so the corpus is conditioned on that terminology and cannot support a comparison between self-medication and competing accounts. The four analytic-stance categories were retained but renamed framing categories, recording how a source characterises the relationship rather than evidence of benefit, and the evidence-strength verdict column and all derived rankings were removed.

Amendment 2 (re-screening). Every included source was re-screened against the version 1.0 population and concept criteria using retrieved abstracts, publication metadata and full text where available. Fifteen sources were found not to meet the criteria and were excluded (three on first re-examination, twelve on the full re-screen), three sources were recoded because their own conclusions did not support self-medication, and substance tags were removed from three sources in which a substance appeared only as a covariate or route of administration. The re-screen was performed by the same single reviewer with large-language-model assistance and does not constitute independent duplicate screening.
