# Supplementary material for: Self-medication of attention-deficit/hyperactivity disorder symptoms with psychoactive substances in adults: a scoping review of how symptom-relief motives are framed and operationalised across substances
Source: Front Psychiatry. 2026 Sep 30;17:1966206. doi: 10.3389/fpsyt.2026.1966206 (PMC13662167; doi:10.3389/fpsyt.2026.1966206)
Supplement: Supplementary file 2 [file Table2.docx]

**Supplementary Material**

**Self-medication of attention-deficit/hyperactivity disorder symptoms with psychoactive substances in adults: a scoping review of how symptom-relief motives are framed and operationalised across substances**

**Supplementary Table 1. Characteristics of included sources.**

Supplementary Table 1 summarises all 109 included sources, grouped by framing category and then by substance. For each source it gives the authors (up to six, then et al.) and year, design or document type, the substance(s) addressed, and the framing category assigned during charting. Fields that were unreported or not applicable across the corpus (formal outcome-evaluation approach, study duration, and setting) have been omitted; sample sizes and countries are given in the narrative and audit ledger where reported.

| **Source (authors, year)** | **Design / document type** | **Substance(s)** | **Framing category** |
| --- | --- | --- | --- |
| Carroll KM, Rounsaville BJ (1993) | Neurobiological/imaging | Alcohol; Cocaine | Self-medication-framed |
| Hooten M, Silverstein S, Daniulaityte R (2025) | Cross-sectional | Amph/meth; Opioids | Self-medication-framed |
| Aloumanis J, Chen S, Allen JH, Yu CC, Nixon SJ, Elton A (2026) | Cross-sectional | Cannabis | Self-medication-framed |
| Mavaddat H, Fouladi A, Kashani AS, Khayatan I, Andarzbaksh K, Razavi SM, et al (2025) | Review | Cannabis | Self-medication-framed |
| de Bode N, Kroon E, Sznitman SR, Cousijn J (2025) | Syst. review/MA | Cannabis | Self-medication-framed |
| Müller-Vahl KR (2024) | Review | Cannabis | Self-medication-framed |
| Dhamija D, Bello AO, Khan AA, Gutlapalli SD, Sohail M, Patel PA, et al (2023) | Syst. review/MA | Cannabis | Self-medication-framed |
| Gujska JH, Silczuk A, Madejek R, Szulc A (2023) | Review | Cannabis | Self-medication-framed |
| Mansell H, Quinn D, Kelly LE, Alcorn J (2022) | Qualitative | Cannabis | Self-medication-framed |
| The use of cannabinoids in the treatment of ADHD: a review (2022) | Review | Cannabis | Self-medication-framed |
| Stueber A, Cuttler C (2021) | Cross-sectional | Cannabis | Self-medication-framed |
| Elucidating the Nature of the Links Between Cannabis Use and ADHD (2021) | Cross-sectional | Cannabis | Self-medication-framed |
| Pedersen W, Sandberg S (2012) | Qualitative | Cannabis | Self-medication-framed |
| Skutle A, Bu ETH, Jellestad FK, van Emmerik-van Oortmerssen K, Dom G, Verspreet S, et al (2015) | Qualitative | Cannabis; Alcohol; Non-Rx stim. | Self-medication-framed |
| Grundmann O, Veltri CA, Morcos S, Smith KE, Singh D, Corazza O, et al (2023) | Cross-sectional | Cannabis; Kratom; Benzodiazepines | Self-medication-framed |
| Microdosing psychedelics in the treatment of ADHD and comorbid disorders (2024) | Cross-sectional | Psychedelics | Self-medication-framed |
| Psilocybin as a First-Line Treatment of ADHD in Adult Populations (2024) | Review | Psychedelics | Self-medication-framed |
| Haijen ECHM, Hurks PPM, Kuypers KPC (2022) | Experimental / trial | Psychedelics | Self-medication-framed |
| Hutten NRPW, Mason NL, Dolder PC, Kuypers KPC (2019) | Experimental / trial | Psychedelics | Self-medication-framed |
| Self-medication with Novel Psychoactive Substances (NPS): a Systematic Review (2023) | Syst. review/MA | Psychedelics; Benzos | Self-medication-framed |
| Carli G, Cavicchioli M, Martini AL, Bruscoli M, Manfredi A, Presotto L, et al (2023) | Neurobiological/imaging | Cocaine | Self-medication-framed |
| Cardullo S, Gómez Pérez LJ, Cuppone D, Sarlo M, Cellini N, Terraneo A, et al (2021) | Observational / comparative | Cocaine | Self-medication-framed |
| Cocores JA, Davies RK, Mueller PS, Gold MS (1987) | Case report/series | Cocaine | Self-medication-framed |
| Weiss RD, Mirin SM (1986) | Cross-sectional | Cocaine | Self-medication-framed |
| Manni C, Cipollone G, Pallucchini A, Maremmani AGI, Perugi G, Maremmani I (2019) | Cross-sectional | Cocaine; Non-Rx stim. | Self-medication-framed |
| Odell AP, Reynolds GL, Fisher DG, Huckabay LM, Pedersen WC, Xandre P, et al (2017) | Cross-sectional | Cocaine; Non-Rx stim.; Amph/meth | Self-medication-framed |
| Hornig M (1998) | Neurobiological/imaging | General/multiple substances | Self-medication-framed |
| Bath R, Bucholz T, Buros AF, Singh D, Smith KE, Veltri CA, et al (2020) | Cross-sectional | Kratom | Self-medication-framed |
| Taylor MR, Carrasco K, Carrasco A, Basu A (2022) | Review | Nicotine | Self-medication-framed |
| Besson M, Forget B (2016) | Review | Nicotine | Self-medication-framed |
| Kutlu MG, Parikh V, Gould TJ (2015) | Review | Nicotine | Self-medication-framed |
| Symmes A, Winters KC, Fahnhorst T, Botzet A, Lee S, August G, et al (2015) | Cohort | Nicotine | Self-medication-framed |
| Ashare RL, Hawk LW (2011) | Experimental / trial | Nicotine | Self-medication-framed |
| Pinkhardt EH, Kassubek J, Brummer D, Koelch M, Ludolph AC, Fegert JM, et al (2009) | Cross-sectional | Nicotine | Self-medication-framed |
| Gehricke JG, Hong N, Whalen CK, Steinhoff K, Wigal TL (2009) | Experimental / trial | Nicotine | Self-medication-framed |
| Poltavski DV, Petros T (2006) | Experimental / trial | Nicotine | Self-medication-framed |
| Gehricke JG, Whalen CK, Jamner LD, Wigal TL, Steinhoff K (2006) | Experimental / trial | Nicotine | Self-medication-framed |
| Lerman C, Audrain J, Tercyak K, Hawk LW, Bush A, Crystal-Mansour S, et al (2001) | Commentary / hypothesis | Nicotine | Self-medication-framed |
| Coger RW, Moe KL, Serafetinides EA (1996) | Qualitative | Nicotine | Self-medication-framed |
| Ágoston C, Urbán R, Horváth Z, van den Brink W, Demetrovics Z (2022) | Cross-sectional | Caffeine | Misuse- or shared-liability-framed |
| Strada L, Korteling S, Vergeer M, Oomen P (2025) | Cross-sectional | Cannabis; Non-Rx stim. | Self-medication-framed |
| Gehricke JG, Hong N, Wigal TL, Chan V, Doan A (2011) | Cross-sectional | Nicotine; Non-Rx stim. | Self-medication-framed |
| Conners CK, Levin ED, Sparrow E, Hinton SC, Erhardt D, Meck WH, et al (1996) | Experimental / trial | Nicotine; Non-Rx stim.; Amph/meth | Self-medication-framed |
| Holborn T, Schifano F, Smith E, Deluca P (2025) | Qualitative | Non-Rx stim. | Self-medication-framed |
| Holborn T, Schifano F, Deluca P (2023) | Qualitative | Non-Rx stim. | Self-medication-framed |
| Volkow ND, Swanson JM (2003) | Neurobiological/imaging | Non-Rx stim. | Self-medication-framed |
| Fallah G, Moudi S, Hamidia A, Bijani A (2018) | Cross-sectional | Non-Rx stim.; Amph/meth | Self-medication-framed |
| Zaami S, Tagliabracci A, Berretta P, Busardò FP, Marinelli E (2020) | Review | Non-Rx stim.; Amph/meth; Classic psychedelics; Modafinil | Self-medication-framed |
| Miller AHF, Krotulski AJ, Walton SE, Dulaney AR, Frolichstein AP, Dusek HL, et al (2025) | Case report/series | Kratom | Self-medication-framed |
| Novak SP, Kroutil LA, Williams RL, Van Brunt DL (2007) | Cross-sectional | Alcohol; Non-Rx stim. | Mixed |
| Matsumoto T (2013) | Review | Amph/meth; Inhalants | Mixed |
| Vieira JL, Coelho SG, Snaychuk LA, Tabri N, Dawson SJ, Hodgins DC, et al (2024) | Cross-sectional | Cannabis; Alcohol | Mixed |
| Young S, González RA, Wolff K, Mutch L, Malet-Lambert I, Gudjonsson GH (2017) | Review | General/multiple substances | Mixed |
| van Amsterdam J, van der Velde B, Schulte M, van den Brink W (2017) | Syst. review/MA | Nicotine | Mixed |
| Sousa NO, Grevet EH, Salgado CA, Silva KL, Victor MM, Karam RG, et al (2010) | Qualitative | Nicotine | Mixed |
| Glass K, Flory K (2010) | Review | Nicotine | Mixed |
| Culpepper L (2013) | Review | Non-Rx stim. | Mixed |
| Varga MD (2012) | Review | Non-Rx stim. | Mixed |
| Peterkin AL, Crone CC, Sheridan MJ, Wise TN (2010) | Cross-sectional | Non-Rx stim. | Mixed |
| Rabiner DL, Anastopoulos AD, Costello EJ, Hoyle RH, McCabe SE, Swartzwelder HS (2008) | Cross-sectional | Non-Rx stim. | Mixed |
| Lembke A (2012) | Review | Alcohol; Amph/meth | Misuse- or shared-liability-framed |
| Upadhyaya HP (2007) | Cross-sectional | Alcohol; Non-Rx stim. | Misuse- or shared-liability-framed |
| Hernandez M, Levin FR (2022) | Review | Cannabis | Misuse- or shared-liability-framed |
| Young S, Sedgwick O (2015) | Review | General/multiple substances | Misuse- or shared-liability-framed |
| Natter J, Michel B (2020) | Qualitative (content analysis) | Memantine | Misuse- or shared-liability-framed |
| Liebrenz M, Frei A, Fisher CE, Gamma A, Buadze A, Eich D (2014) | Qualitative | Nicotine | Misuse- or shared-liability-framed |
| Fairman RT, Vu M, Haardörfer R, Windle M, Berg CJ (2020) | Cross-sectional | Non-Rx stim. | Misuse- or shared-liability-framed |
| Chamakalayil S, Strasser J, Vogel M, Brand S, Walter M, Dürsteler KM (2021) | Review | Non-Rx stim. | Misuse- or shared-liability-framed |
| Schepis TS, Buckner JD, Klare DL, Wade LR, Benedetto N (2020) | Intensive longitudinal (EMA/daily diary) | Non-Rx stim. | Misuse- or shared-liability-framed |
| Benson K, Woodlief DT, Flory K, Siceloff ER, Coleman K, Lamont A (2018) | Cross-sectional | Non-Rx stim. | Misuse- or shared-liability-framed |
| Bavarian N, Flay BR, Ketcham PL, Smit E (2013) | Cross-sectional | Non-Rx stim. | Misuse- or shared-liability-framed |
| Upadhyaya HP, Kroutil LA, Deas D, Durell TM, Van Brunt DL, Novak SP (2010) | Cross-sectional | Non-Rx stim. | Misuse- or shared-liability-framed |
| Wilens TE, Adler LA, Adams J, Sgambati S, Rotrosen J, Sawtelle R, et al (2008) | Syst. review/MA | Non-Rx stim. | Misuse- or shared-liability-framed |
| Wilens TE (2004) | Review | Non-Rx stim. | Misuse- or shared-liability-framed |
| Rockhill KM, Olson R, Dart RC, Iwanicki JL, Black JC (2023) | Cross-sectional | Non-Rx stim.; Amph/meth; Opioids | Misuse- or shared-liability-framed |
| ADHD stimulant medication misuse and considerations for prescribing practice (2021) | Review | Non-Rx stim.; Caffeine | Misuse- or shared-liability-framed |
| Margherio SM, Kang S, Kennedy TM, Pedersen SL, Gnagy E, Molina BSG, et al (2026) | Cohort | Alcohol | Neutral / descriptive |
| Sherman AL, Stamates AL, Weyandt L (2025) | Cross-sectional | Alcohol | Neutral / descriptive |
| Grazioli VS, Gmel G, Rougemont-Bücking A, Baggio S, Daeppen JB, Studer J (2019) | Cohort | Alcohol | Neutral / descriptive |
| Wilens TE (1998) | Review | Alcohol | Neutral / descriptive |
| Young S, González RA, Wolff K, Xenitidis K, Mutch L, Malet-Lambert I, et al (2017) | Qualitative | Alcohol; Cocaine; Opioids; Amph/meth | Neutral / descriptive |
| Thurn D, Riedner A, Wolstein J (2020) | Qualitative | Amph/meth | Misuse- or shared-liability-framed |
| Broderick P, Benjamin AB (2004) | Review | Caffeine | Neutral / descriptive |
| Goldstein AL, Shifrin A, Katz JL, Iu LK, Kofler D (2021) | Cross-sectional | Cannabis | Neutral / descriptive |
| Stevens AK, Gunn RL, Jackson KM, Borsari B, Metrik J (2020) | Cohort | Cannabis | Neutral / descriptive |
| Mitchell JT, Sweitzer MM, Tunno AM, Kollins SH, McClernon FJ (2016) | Qualitative | Cannabis | Neutral / descriptive |
| From badness to illness: Medical cannabis and self-diagnosed ADHD (2014) | Qualitative | Cannabis | Neutral / descriptive |
| Loflin M, Earleywine M, De Leo J, Hobkirk A (2013) | Cross-sectional | Cannabis | Neutral / descriptive |
| Taubin D, Oddo LE, Bounoua N, Bui HNT, Murphy JG, Chronis-Tuscano A (2025) | Intensive longitudinal (EMA/daily diary) | Cannabis; Alcohol | Neutral / descriptive |
| Mosandl CF, Baltes-Flückiger L, Kronschnabel J, Meyer M, Guessoum A, Herrmann O, et al (2024) | Cross-sectional | Cannabis; Alcohol | Neutral / descriptive |
| Swanson JM, Wigal T, Jensen PS, Mitchell JT, Weisner TS, Murray D, et al (2017) | Qualitative | Cannabis; Alcohol | Neutral / descriptive |
| Peles E, Schreiber S, Linzy S, Domani Y, Adelson M (2014) | Neurobiological/imaging | Cannabis; Cocaine; Opioids; Non-Rx stim.; Benzodiazepines; Amph/meth | Neutral / descriptive |
| Lowden DS, Wong JHK, Harty SC (2025) | Cross-sectional | Cannabis; Non-Rx stim. | Neutral / descriptive |
| Hupli AMM (2018) | Review | Cannabis; Non-Rx stim. | Neutral / descriptive |
| Young S, Abbasian C, Al-Attar Z, Branney P, Colley B, Cortese S, et al (2023) | Review | General/multiple substances | Neutral / descriptive |
| Young S, Woodhouse E (2020) | Qualitative | General/multiple substances | Neutral / descriptive |
| Wilens TE (2004) | Cross-sectional | General/multiple substances | Neutral / descriptive |
| Wilson JJ, Levin FR (2001) | Review | General/multiple substances | Neutral / descriptive |
| Treur JL, Willemsen G, Bartels M, Geels LM, van Beek JH, Huppertz C, et al (2014) | Cross-sectional | Nicotine | Misuse- or shared-liability-framed |
| Gehricke JG, Polzonetti C, Caburian C, Gratton E (2013) | Cross-sectional | Nicotine | Neutral / descriptive |
| Ashor AW (2012) | Cross-sectional | Nicotine | Neutral / descriptive |
| Ohlmeier MD, Peters K, Kordon A, Seifert J, Wildt BT, Wiese B, et al (2007) | Cross-sectional | Nicotine; Alcohol | Neutral / descriptive |
| Treur JL, Demontis D, Smith GD, Sallis H, Richardson TG, Wiers RW, et al (2019) | Cohort | Nicotine; Cannabis; Alcohol | Neutral / descriptive |
| Upadhyaya HP, Carpenter MJ (2008) | Cross-sectional | Nicotine; Cannabis; Alcohol | Neutral / descriptive |
| Murphy KG, Flory K (2016) | Cross-sectional | Nicotine; Non-Rx stim. | Neutral / descriptive |
| Mitchell JT, McIntyre EM, McClernon FJ, Kollins SH (2013) | Cross-sectional | Nicotine; Non-Rx stim. | Neutral / descriptive |
| Benson K, Flory K (2017) | Cross-sectional | Non-Rx stim. | Neutral / descriptive |
| Hartung CM, Canu WH, Cleveland CS, Lefler EK, Mignogna MJ, Fedele DA, et al (2013) | Cross-sectional | Non-Rx stim. | Neutral / descriptive |
| Moustafa Y, Chauhan M, Rummans TA (2022) | Review | Non-Rx stim.; Amph/meth | Neutral / descriptive |

**Supplementary Table 2. Full electronic search strategies.**

*Structure.* All searches combined a fixed ADHD population block with a concept/substance block using the Boolean form **(ADHD population) AND (substance term(s)) AND (self-medication/motive term(s))**. Because the NCBI E-utilities interface returned no result for the full single combined query, the strategy was executed as one direct self-medication search plus one search per substance family; every executed query, interface and hit count is logged verbatim below and in the audit ledger. OpenAlex, Consensus and ClinicalTrials.gov were searched with the semantic/field queries also listed below.

*Fixed ADHD population block (all PubMed searches).*

`("attention deficit disorder with hyperactivity"[MeSH Terms] OR ADHD[tiab] OR "attention deficit"[tiab] OR hyperkinetic*[tiab] OR "attention-deficit"[tiab])`

*Fixed self-medication / motive block (direct search; a compact variant `"self-medicat*" OR "self-treat*" OR "symptom relief" OR "manage symptoms" OR "alleviate" OR "reduce symptoms" OR "reasons for use" OR "motives" OR "to cope" OR "improve focus" OR "improve concentration"` was AND-combined with each substance block).*

`("self medication"[MeSH Terms] OR "self-medication"[tiab] OR "self-medicating"[tiab] OR "self-medicate"[tiab] OR "self medication"[tiab] OR "self-treatment"[tiab] OR "self-treat"[tiab] OR "symptom relief"[tiab] OR "symptom management"[tiab] OR "alleviate symptoms"[tiab] OR "manage symptoms"[tiab] OR "coping motives"[tiab] OR "reasons for use"[tiab] OR "motives for use"[tiab] OR "motivations for use"[tiab])`

*Substance blocks (PubMed, each AND-combined with the ADHD and motive blocks above).*

- **Nicotine / tobacco:** nicotine[tiab] OR tobacco[tiab] OR smoking[tiab] OR cigarette*[tiab] OR vaping[tiab] (n = 62)
- **Cannabis / cannabinoids:** cannabis[tiab] OR cannabinoid*[tiab] OR marijuana[tiab] OR marihuana[tiab] OR THC[tiab] OR cannabidiol[tiab] OR CBD[tiab] (n = 40)
- **Alcohol:** alcohol[tiab] OR drinking[tiab] OR ethanol[tiab] (n = 40)
- **Cocaine:** cocaine[tiab] OR crack[tiab] (n = 21)
- **Caffeine:** caffeine[tiab] OR "energy drink*"[tiab] OR coffee[tiab] (n = 2)
- **Opioids:** opioid*[tiab] OR heroin[tiab] OR oxycodone[tiab] OR "opiate*"[tiab] (n = 16)
- **MDMA:** MDMA[tiab] OR ecstasy[tiab] OR "3,4-methylenedioxymethamphetamine"[tiab] (n = 1)
- **Psychedelics:** psychedelic*[tiab] OR psilocybin[tiab] OR LSD[tiab] OR "lysergic"[tiab] OR microdos*[tiab] OR ayahuasca[tiab] OR mescaline[tiab] (n = 3)
- **Ketamine:** ketamine[tiab] OR esketamine[tiab] (n = 2)
- **Benzodiazepines:** benzodiazepine*[tiab] OR diazepam[tiab] OR alprazolam[tiab] (n = 4)
- **GHB / inhalants / kratom / novel:** GHB[tiab] OR inhalant*[tiab] OR kratom[tiab] OR "nitrous oxide"[tiab] OR modafinil[tiab] (n = 8)
- **Prescription stimulants (methylphenidate, amphetamine):** methylphenidate[tiab] OR amphetamine*[tiab] OR "prescription stimulant*"[tiab] (n = 36)
- **Study/smart drugs; non-medical/diverted use:** "study drug*"[tiab] OR "smart drug*"[tiab] OR "nonmedical"[tiab] OR "non-medical"[tiab] OR diverted[tiab] OR misuse[tiab] (n = 26)

*Supplementary interfaces.* The PubMed strategy above (one direct-concept search plus 13 substance searches, 14 in total) was complemented by relevance-ranked and field searches on four further interfaces. Each executed query is reproduced verbatim below; platform conventions are: OpenAlex — OpenAlex REST API (relevance-ranked; top 50/query captured); Consensus — consensus.app aggregator (relevance-ranked; ~20/query); ClinicalTrials.gov — ClinicalTrials.gov API (condition + intervention fields); OpenAlex preprint — OpenAlex REST API, preprint filter (top 40/query). Reported record counts are the interface totals returned; the eligible set was determined at screening (see Fig. 1). The full machine-readable log of all 38 searches, with dates and per-source identifiers, is held in the audit ledger (charting.json).

| **#** | **Interface** | **Query (verbatim)** | **Records** |
| --- | --- | --- | --- |
| 1 | OpenAlex | ADHD self-medication substance use adults | 19939 |
| 2 | OpenAlex | attention deficit hyperactivity disorder cannabis self-medication | 5249 |
| 3 | OpenAlex | ADHD nicotine smoking self-medication | 2837 |
| 4 | OpenAlex | ADHD stimulant misuse self-medication motives | 568 |
| 5 | OpenAlex | ADHD ketamine self-medication | 1884 |
| 6 | OpenAlex | ADHD psychedelics microdosing self-medication | 101 |
| 7 | OpenAlex | ADHD alcohol self-medication motives adults | 1607 |
| 8 | OpenAlex | ADHD cocaine self-medication hypothesis | 2824 |
| 9 | Consensus | ADHD self-medication with substances in adults | 20 |
| 10 | Consensus | cannabis self-medication ADHD symptoms adults | 20 |
| 11 | Consensus | nicotine smoking self-medication ADHD | 20 |
| 12 | Consensus | self-medication hypothesis ADHD substance use disorder | 20 |
| 13 | Consensus | ketamine ADHD self-medication | 20 |
| 14 | Consensus | psychedelics microdosing ADHD self-treatment | 20 |
| 15 | Consensus | alcohol self-medication ADHD adults | 20 |
| 16 | Consensus | cocaine self-medication ADHD | 20 |
| 17 | Consensus | prescription stimulant misuse ADHD self-medication | 18 |
| 18 | ClinicalTrials.gov | cond=attention deficit hyperactivity disorder; interv=cannabis OR cannabinoid OR THC | 12 |
| 19 | ClinicalTrials.gov | cond=attention deficit hyperactivity disorder; interv=ketamine | 1 |
| 20 | ClinicalTrials.gov | cond=attention deficit hyperactivity disorder; interv=psilocybin OR LSD OR psychedelic | 1 |
| 21 | ClinicalTrials.gov | cond=attention deficit hyperactivity disorder; interv=nicotine | 12 |
| 22 | OpenAlex-preprint | ADHD self-medication cannabis [preprint] | 246 |
| 23 | OpenAlex-preprint | ADHD self-medication ketamine psychedelic [preprint] | 22 |
| 24 | OpenAlex-preprint | ADHD self-medication substance adults [preprint] | 962 |

**Supplementary Table 3. Completed PRISMA-ScR checklist.**

| **Section** | **Item** | **PRISMA-ScR checklist item** | **Reported in** |
| --- | --- | --- | --- |
| Title | 1 | Identify the report as a scoping review. | Title |
| Abstract | 2 | Structured summary (background, objectives, eligibility, sources, charting, results, conclusions). | Structured abstract |
| Introduction | 3 | Rationale; why a scoping review. | Introduction (final paragraph) |
|  | 4 | Questions and objectives with PCC elements. | Aim; Method Stage 1 |
| Methods | 5 | Protocol and registration. | Method (opening); Declarations — Protocol |
|  | 6 | Eligibility criteria with rationale. | Method Stage 1 (PCC); Stage 2 (language/date) |
|  | 7 | Information sources; coverage dates; last search date. | Method Stage 2 |
|  | 8 | Full electronic search strategy for ≥one database. | Method; Supplementary Table 2 |
|  | 9 | Selection of sources of evidence (screening/eligibility). | Method Stage 3; Fig. 1 |
|  | 10 | Data charting process; piloting; independence. | Method Stage 4 |
|  | 11 | Data items: variables and definitions. | Method Stage 4; Stage 5 |
|  | 12 | Critical appraisal (optional). | Not applicable — no critical appraisal undertaken (Method Stage 4) |
|  | 13 | Synthesis of results. | Method Stage 5 |
| Results | 14 | Selection of sources of evidence (numbers, exclusions, flow). | Results — Selection; Fig. 1 |
|  | 15 | Characteristics of sources of evidence with citations. | Results — Overview; Supplementary Table 1 |
|  | 16 | Critical appraisal within sources (optional). | Not applicable — no critical appraisal undertaken |
|  | 17 | Results of individual sources of evidence. | Results — Substance-by-substance map; audit ledger |
|  | 18 | Synthesis of results. | Results — Substance-by-substance map; Fig. 2 |
| Discussion | 19 | Summary of evidence; link to questions; relevance. | Discussion — Summary; Why the account varies |
|  | 20 | Limitations of the scoping review process. | Discussion — Strengths and limitations |
|  | 21 | Conclusions; implications and next steps. | Conclusion |
| Funding | 22 | Funding of included sources and of the review. | Funding of included sources: not charted (Method); funding of this review: Declarations — Funding |

**Supplementary Table 4. Full-text exclusions with reasons.**

| **#** | **Citation** | **Reason for exclusion** |
| --- | --- | --- |
| 1 | Transcranial direct current stimulation (tDCS) combined with cognitive training in adolescent boys with ADHD: a double-blind, randomised, sham-controlled trial. 2020. doi:10.1101/2020.12.07.20245456 | Not substance self-medication / not adult |
| 2 | “It has to be fixed”: a qualitative inquiry into perceived ADHD behaviour among affected individuals and parents in Western Australia. 2016. PMID:27101981 doi:10.1186/s12913-016-1399-1 | Concerns prescribed treatment, not self-medication |
| 3 | Attention deficit hyperactivity disorder and obsessive-compulsive disorder among former heroin addicts currently in methadone maintenance treatment. 2012. PMID:22796643 doi:10.1159/000336219 | Comorbidity prevalence only; no self-medication concept |
| 4 | Activation instead of blocking mesolimbic dopaminergic reward circuitry is a preferred modality in the long term treatment of reward deficiency syndrome (RDS): a commentary. 2008. PMID:19014506 doi:10.1186/1742-4682-5-24 | Not ADHD-specific |
| 5 | Regulation of dopamine and MPP+ transport by catecholamine transporters. 2004. PMID:15164608 | Mechanistic/in-vitro; concept peripheral |
| 6 | Cocaine addiction as a neurological disorder: implications for treatment. 1996. PMID:8809851 | Substance-induced deficits, not ADHD self-medication |
| 7 | Predictors of Depression and Anxiety Among Self-Medicating Autistic Adults. 2025. doi:10.1177/27546330251344453 | Concerns self-medication in autistic adults, not an adult ADHD population |
| 8 | Horner BR, Scheibe KE. Prevalence and implications of ADHD among adolescents in treatment for substance abuse. 1997. PMID:9204671 doi:10.1097/00004583-199701000-00014 | Adolescent sample, not an adult or predominantly-adult population |
| 9 | Spasova V, Mehmood S, Minhas A, et al. Impact of nicotine on cognition in patients with schizophrenia: a narrative review. Cureus. 2022. doi:10.7759/cureus.24306 | Population is patients with schizophrenia, not adults with ADHD |
| 10 | McNaughton N, Glue P. Ketamine and neuroticism: a double-hit hypothesis of internalizing disorders. Personal Neurosci. 2020. doi:10.1017/pen.2020.2 | Does not concern ADHD; hypothesis paper on neuroticism and internalising disorders |
| 11 | Black N, Stockings E, Campbell G, et al. Cannabinoids for the treatment of mental disorders and symptoms of mental disorders: a systematic review and meta-analysis. Lancet Psychiatry. 2019. doi:10.1016/S2215-0366(19)30401-8 | Systematic review of formal cannabinoid treatment efficacy, not self-medication behaviour (concept criterion) |
| 12 | Cooper RE, Williams E, Seegobin S, Tye C, Kuntsi J, Asherson P (2017) (re-screened on revision) | Formal cannabinoid treatment trial, not self-medication |
| 13 | Mansell H, Quinn D, Kelly LE, Szafron M, Alcorn J (2021) (re-screened on revision) | Protocol only (no data); ages 12-25 |
| 14 | Silva N, Szobot CM, Shih MC, Hoexter MQ, Anselmi CE, Pechansky F, et al (2014) (re-screened on revision) | Adolescent sample; not adult |
| 15 | Bizzarri JV, Rucci P, Sbrana A, Gonnelli C, Massei GJ, Ravani L, et al (2006) (re-screened on revision) | Not an ADHD population (ADHD as vulnerability-domain score only) |
| 16 | Wilens TE, Adamson J, Sgambati S, Whitley J, Santry A, Monuteaux MC, et al (2007) (re-screened on revision) | Adolescent/young-adult sample; not predominantly adult |
| 17 | Desantis A, Noar SM, Webb EM (2009) (re-screened on revision) | General-population sample; ADHD not ascertained |
| 18 | DeSantis A, Noar SM, Webb EM (2010) (re-screened on revision) | General-population sample; ADHD not ascertained |
| 19 | Dupont RL, Coleman JJ, Bucher RH, Wilford BB (2008) (re-screened on revision) | General-population sample; ADHD not ascertained |
| 20 | McCabe SE, Figueroa O, McCabe VV, Schepis TS, Schulenberg JE, Veliz PT, et al (2023) (re-screened on revision) | Adolescent sample; not adult |
| 21 | Fuermaier ABM, Tucha O, Koerts J, Tucha L, Thome J, Faltraco F (2021) (re-screened on revision) | General-population sample; ADHD not ascertained |
| 22 | Riegler A, Völkl-Kernstock S, Lesch O, Walter H, Skala K (2017) (re-screened on revision) | Adolescent sample; not adult |
| 23 | Teter CJ, McCabe SE, LaGrange K, Cranford JA, Boyd CJ (2006) (re-screened on revision) | General-population sample; ADHD not ascertained |
